# Clinical Behavior of Triple Negative Breast Cancer in a Cohort of Latin American Women

**DOI:** 10.7759/cureus.4963

**Published:** 2019-06-21

**Authors:** Sandra Diaz Casas, Eder Lancheros García, Andrés Sanchéz Campo, Ricardo Sanchez Pedraza, Vivian Roman Vasquez, Sara D Mendoza, Javier Angel Aristizabal, Carlos Lehmann Mosquera, Carlos Duarte Torres, Juan C Vergel

**Affiliations:** 1 Breast and Soft Tissue Clinic, Instituto Nacional de Cancerologia, Bogotá D.C., COL; 2 Breast and Soft Tissue Clinic, Clinica De Occidente, Bogotá D.C., COL; 3 Office of the Deputy Director for Research, Epidemiological Surveillance, Promotion and Prevention, Instituto Nacional de Cancerologia, Bogotá D.C., COL; 4 Breast Surgery, Fundacion Valle Del Lili, Bogotá D.C., COL; 5 Oncology, Instituto Nacional de Cancerologia, Bogotá D.C., COL

**Keywords:** breast neoplasm, cohort, survival, chemotherapy, surgery

## Abstract

Introduction: Breast cancer is a worldwide public health problem. In Colombia, there are 13,000 new cases, having the highest incidence and mortality among cancers. This article describes the clinical behavior of patients with triple negative breast cancer (TNBC) treated at the National Cancer Institute (NCI) in Bogota, Colombia.

Methods: A historical cohort and analytical study that included elderly patients diagnosed with TNBC treated at the National Cancer Institute Functional Breast Cancer Unit (NCI-FBCU) was conducted.

Results: Of the 1,066 patients registered in the unit from September 1st 2013 to December 31st 2016: 146 (13.7 %) had triple negative tumors. The average age was 57.3 years; 61% of patients had locally advanced tumors. The majority of patients received neoadjuvant chemotherapy as their first treatment (69.1%), and in 41.2% of the cases platinum was added to the chemotherapy regimen. The most common surgery conducted was modified radical mastectomy in 57.8% of cases. The pathological complete response (pCR) (Chevallier 1 and 2) was reached in 22.6% and, in this group of patients, a greater overall survival (OS) was found [hazard ratio (HR) 0.08, 95% CI 0.01-0.63; p = 0.016]. Progression of the disease occurred in 36.5% of cases, being lungs the most frequent location (44.4%). The death incidence rate was 1.21 deaths per 100 patients/month. The median event-free survival (EFS) was 18.2 months.

Conclusion: TNBC occurs in Latin American women at advanced clinical stages with aggressive clinical behavior, with lower OS rates, and higher risk of metastasis compared to other molecular subtypes.

## Introduction

Breast cancer is a worldwide public health problem with 2.1 million new cases diagnosed in 2018, according to data from the latest GLOBOCAN update. It ranks first in incidence of female cancer worldwide with 24.2%, and is the leading cause of death from cancer in women (15%) [1].

In Colombia, the number of new cases of breast cancer in 2017 accounted for 13.1% of all cancers in both sexes; being the most common cancer in our country with 13,380 women diagnosed per year. It also ranks first in mortality accounting for 15.9% of all cases [1].

The National Cancer Institute (NCI) is the most important reference cancer center in Colombia. At the National Cancer Institute Functional Breast Cancer Unit (NCI-FBCU), patients who were admitted to the NCI for the first time with a diagnosis of cancer and who have not yet received any type of previous treatment, are conducted through diagnostic services of the hospital and then introduced at a meeting where a Breast Surgeon and a Clinical Oncologist participate in order to define the patients’ treatments; in addition, the coordinator of NCI-FBCU (a Nurse Oncologist) is responsible for ensuring management, monitoring, and completion of the database where treatments, dates, and outcomes are recorded.

From September 1st 2013 to December 31st 2016, 1,066 new cases of breast cancer were admitted to the functional unit, of which 146 were triple negative tumors (TNBC), accounting for 13.7% of all cases diagnosed in that period of time.

Current scientific evidence establishes that TNBC accounts for 15% of all cancers diagnosed and affects mainly women who are Hispanic, Afro-American, pre-menopausal, or carriers of BRCA gene mutations [2].

A retrospective cohort study conducted in 2008 at the MD Anderson Cancer Center, included 491 patients with breast cancer and documented a mutation in the BRCA gene in 17.5% of the cases; of these, 57.6% were TNBC, the most frequent mutation in this subgroup of patients was in the BRCA1 gene with 57.1% of cases, in the BRCA2 with 23.3% of the cases, and 13.8% were nonmutated [3].

Triple negative breast cancer is a tumor that expresses less than 1% estrogens and progestogens, and is HER2-negative; it is a subtype with greater aggressiveness in its appearance, associated with greater tumor size, high histological grade and frequently with lymph node involvement [2], because of this it has a high risk of distant metastasis, especially to lungs, brain, and liver. The risk of recurrence in the first three years after starting treatment is greater compared to other molecular subtypes of breast cancer [4-5].

Triple negative breast cancer is a heterogeneous disease by itself. In 2011, Lehmann et al. identified six specific subtypes by using gene expression profiles through microarray technology. These subtypes are: basal-like 1 (BL1), basal-like 2 (BL2), immunomodulatory (IM), mesenchymal (M), mesenchymal stem cell (MSL), and luminal androgen receptor (LAR) [6].

Researchers concluded that each subtype described different clinical behavior, sensitivity to chemotherapy, and overall survival (OS) [6].

Parallel to this classification, Burstein et al. developed a DNA and RNA sequence in 200 tumors with TNBC, by which they identified four subtypes: luminal androgens receptor, mesenchymal, basal immunosuppressant, and basal immunoactive [7].

In this classification, the basal-like immunosuppressant subtype has the worst prognosis, and the basal-like immunoactive has the best prognosis, in terms of OS and event-free survival (EFS) [7].

The purpose of this article is to describe the clinical behavior of TNBC patients who are treated at the NCI-FBCU during the period described above.

## Materials and methods

An observational, analytical, historical, and cohort study was conducted. The cohort was defined as “patients with a confirmed diagnosis of TNBC, who were admitted into NCI-FCBU in Bogota, Colombia, and received their entire treatment during a period of three years (September 1st 2013 until December 31st 2016).” Patients with luminal breast cancer, HER2-positive, and those who did not receive full treatment within the NCI-FCBU, were excluded. This work was approved by the NCI Institutional Ethics Committee.

Database from the NCI-FBCU and records from the NCI digital clinical history (SAP®, SAP SE, Walldorf, Germany), were used as a source of information for the selection of patients. Two co-researchers, specialists in breast surgery, extracted data. We reviewed treatment records, start date and follow-up, as well as one-by-one records of each SAP® patient's clinical history, on the following aspects: registered demographic data, pathology report of the biopsy and surgical specimen, chemotherapy records, radiotherapy records, follow-up consultation from the Breast and Soft Tissue Clinic, Clinical Oncology, Radiotherapy, Palliative Care, and Emergency.

The clinical response was assessed with the scale of the World Health Organization (WHO) [8], and the pathological response with Chevallier criteria [9].

The variables included in this study were: age, health assurance system, first-degree family history with breast cancer, body mass index, menopausal status, pathological characteristics of the biopsy, clinical stage of the disease, initial treatment received, clinical response obtained according to the WHO scale, neoadjuvant chemotherapy regimen administered, surgery performed, pathological response according to Chevallier criteria, use of adjuvant radiotherapy, localization an treatment of recurrence, and follow-up [EFS, progression-free survival (PFS) and OS].

Using absolute and relative frequency measures, a descriptive analysis of the categorical and nominal variables was carried out. With regard to continuous variables, measures of central tendency and measures of dispersion were used.

The clinical behavior of patients with TNBC was assessed, and the proportion of patients who received cancer treatment, their response to neoadjuvant chemotherapy, and percentage of their pCR were calculated. The connection between categorical variables was assessed by using Fisher's exact tests.

Survival analyses for the corresponding events were quantified: death from any cause, event-free period, and progression-free period. For EFS and OS, the date of surgical procedure was taken as the time of admission to the cohort. In the case of PFS, the date of the latest cycle of primary chemotherapy was taken as the time of admission to the cohort. Events were taken as numerators to estimate incidence density rates, taking as denominator the total time contributed by patients of the cohort.

These rates were estimated taking into account the risk of bias due to the presence of differential follow-up. Incidence estimators were reported with 95% confidence intervals. Survival functions were estimated for each of the three outcomes, by using the Kaplan-Meier method. These functions took into account the loss of follow-up, handled as censors on the right.

In addition, log-rank test was used to compare survival functions, and significance levels of 5% were used for all hypothesis tests.

Statistical analysis of data was performed using the Stata Statistical Software: Release 12 (StataCorp 2011, StataCorp LP, College Station, TX), licensed for the NCI.

## Results

Of the 1,066 patients registered in the NCI-FBCU from September 2013 to December 2016, 146 (13.7%) had TNBC; 23 patients (15.7%) were excluded due to the following reasons: nine had received some type of treatment outside the NCI; eight died before starting treatment; three patients had tumors with overexpression of HER2; two patients rejected the treatment; and one received incomplete treatment. This analysis included 123 patients: most of them belong to the subsidized health system (59.3%, n = 73); their average age was 57.3 (22-88) years old; the majority was postmenopausal (73.2%, n = 90); a genetic study was ordered to 45 patients (36.5%) and it was conducted in 32 of them; most had complete gene sequencing (59.4%, n = 19). Regarding clinical stage, 61.0% (n = 75) of cases were locally advanced tumors, being stage IIIB (35.0%, n = 43) the most frequent. 18.7% (n = 23) of the patients in this series of cases were admitted with metastatic disease (Table 1).

**Table 1:** Characteristics of patients diagnosed with TNBC at the NCI-FBCU. TNBC, Triple negative breast cancer; NCI-FBCU, National Cancer Institute Functional Breast Cancer Unit.

Characteristics	%	N
Type of health assurance system		
Subsidized	59.3	73
Contributive	40.7	50
Age (years)		57.3
Antecedent of breast cancer on first degree family		
No	79.7	98
Yes	9.8	12
No Data	10.6	13
Body mass index (kg/m^2^)		
Normal (18.1–25)	37.4	46
Overweight (25.1–29.9)	45.5	56
Class I obesity (30–34.9)	11.4	14
Class II obesity (35–39.9)	4.9	6
Morbid obesity (>40)	0.8	1
Genetic study		
Requested test	36.5	45
Performed test	26.0	32
BRCA 1/2 complete sequencing	59.4	19
Colombia profile	34.4	11
Others	6.2	2
Histological type		
NOS ductal	67.5	83
Pure ductal	19.5	24
Metaplastic	4.9	6
Medullar	1.6	2
Papillary	1.6	2
Others	4.9	6
Differentiation grade		
I	0.8	1
II	18.7	23
III	80.5	99
Lymphovascular invasion		
Absent	18.7	23
Present	4.1	5
No data	77.2	95
Androgen receptor report		
Not reported	99.2	122
Negative	0.8	1
CK report (5/6)		
Not reported	93.5	115
Negative	3.3	4
Positive	3.3	4
Ki 67 (%)		
>60	67.5	83
41–60	15.4	19
21–40	10.6	13
<20	4.1	5
No data	2.4	3
Tumor size (T)		
T1	4.1	5
T2	26.8	33
T3	13.0	16
T4b	52.8	65
T4c	1.6	2
T4d	1.6	2
Lymph node involvement (N)		
N0	26.0	32
N1	26.8	33
N2a	35.8	44
N2b	0.0	0
N3a	1.6	2
N3b	2.4	3
N3c	7.3	9
Metastatic involvement		
M0	81.3	100
M1	18.7	23
Location of metastasis		
Multiple locations	47.8	11
Lungs	47.8	11
Bones	34.8	8
Skin	34.8	8
Nonregional lymph nodes	21.5	5
Liver	17.4	4
CNS	4.3	1
Others	12.9	3
Clinical stage		
I	4.1	5
IIA	16.3	20
IIB	7.3	9
IIIA	13.8	17
IIIB	35.0	43
IIIC	4.9	6
IV	18.7	23

The majority of patients received chemotherapy as initial treatment (87.0%, n = 107); 69.1% (n = 85) had neoadjuvant intention and 17.9% (n = 22) had primary intention, because of the advanced clinical status of most patients. The most used regimen was anthracyclines, cyclophosphamide, and taxanes (AC-T) in 41.2% (n = 35) of patients; and in 41.1% of cases (n = 35) carboplatin was added to the chemotherapy regimen. The most performed surgical procedure was modified radical mastectomy (57.8%, n = 59) of patients. pCR was reported in 22.6% (n = 23) of patients. The group of patients in which carboplatin chemotherapy regimens were included, pCR was found in 11 of the 35 patients (31.4%), without statistical significance (p = 0.13). Of the nine patients with mutation in the BRCA1 and BRCA2 genes, seven had neoadjuvant chemotherapy, and of these, only four received regimens that included platinum, and in addition, only one of these four patients had a pCR. A better OS was found in patients who presented pCR compared to patients who presented residual infiltrative disease in the breast and/or axillary metastatic lymph node involvement in the surgical pathology (Chevallier 3-4) (HR 0.08, 95% CI 0.01-0.63; p = 0.016). The residual tumoral size in the surgical pathology ranged between 0 and 20 cm; 60.8% (n = 62) of patients who were taken into surgery had negative node involvement in the surgical piece.

Regarding the adjuvant, 57.8% (n = 71) of patients in this cohort received radiotherapy; 17.9% (n = 22) received adjuvant chemotherapy. 9.8% (n = 12) of patients of the two previous groups received both treatments sequentially (Table 2).

**Table 2:** Types of treatments administered to patients diagnosed with TNBC at the NCI-FBCU.

Types of treatment	%	n
First treatment received		
Neoadjuvant chemotherapy	69.1	85
Primary chemotherapy	17.9	22
Radical surgery	8.1	10
Conservative surgery	4.1	5
Radiotherapy	0.8	1
Neoadjuvant chemotherapy regimen administered		
Adriamycin-Cyclophosphamide-Taxane (AC-T)	41.2	35
Adriamycin-Cyclophosphamide-Taxane-Carboplatin (AC-TC)	23.5	20
Taxane-Carboplatin (TC)	17.6	15
Adriamycin-Cyclophosphamide (AC)	2.4	2
Others	15.3	13
Primary chemotherapy regimen administered		
Taxane-Carboplatin (TC)	45.5	10
Adriamycin-Cyclophosphamide-Taxane (AC-T)	18.2	4
Adriamycin-Cyclophosphamide-Taxane-Carboplatin (AC-TC)	13.6	3
Adriamycin-Cyclophosphamide (AC)	9.1	2
Others	13.6	3
Type of surgical treatment		
Modified radical mastectomy	57.8	59
Quadrantectomy + axillary dissection	22.5	23
Quadrantectomy + sentinel lymph node	9.8	10
Simple mastectomy + sentinel lymph node	7.8	8
Radical mastectomy	2.0	2
Breast reconstruction		
Not performed	84.6	104
Performed	15.4	19
TRAM	52.6	10
Expander/prosthesis	26.3	5
Latissimus dorsi	15.8	3
Latissimus dorsi + prosthesis	5.1	1
Pathological response to neoadjuvant chemotherapy (Chevallier criteria)		
1	20.6	21
2	2.0	2
3	32.4	33
4	24.5	25
Not applicable	20.6	21
Number of lymph nodes reported in the surgical piece of the axilla		
0 nodes	60.8	62
1–3 nodes	26.5	27
4–10	6.9	7
>10 nodes	4.9	5
Type of adjuvant treatment received		
Exclusive radiotherapy	48	59
Chemotherapy and sequential radiotherapy	9.8	12
Exclusive chemotherapy	8.1	10
None	34.1	42
Adjuvant radiotherapy		
50 Gy	57.7	41
>50 Gy	29.6	21
<50 Gy	12.7	9
Adjuvant chemotherapy		
Adriamycin-Cyclophosphamide (AC)	27.3	6
Adriamycin-Cyclophosphamide-Taxane (AC-T)	22.7	5
Taxane-Carboplatin (TC)	18.2	4
Capecitabine	9	2
Adriamycin-Cyclophosphamide-Taxane-Carboplatin (AC-TC)	4.5	1
Taxane	4.5	1
Others	13.6	3

In this cohort, 36.6% (n = 45) presented disease progression mostly with distant metastasis (77.7%, n = 35), being lungs the most frequent location (44.4%, n = 20); and 42.2% (n = 18) of patients had more than two metastatic locations. Local recurrence occurred more frequently in patients taken into radical surgery (13/15; p = 0.35), which was not statistically significant. 11.1% (n = 5) of patients did not receive any type of treatment for the management of recurrence. 87.1% (n = 34) of patients in whom the disease progressed, received some type of treatment; chemotherapy was administered to 25.6% (n = 10); radiotherapy to 38.5% (n = 15); and surgical management of local and/or regional recurrence was performed in 18.0% (n = 7) of cases (Table 3). 

**Table 3:** Location of recurrence within patients diagnosed with TNBC at the NCI-FBCU.

Location of recurrence	%	n
Local	33.3	15
Regional	40.0	15
Distance	71.1	35
Specific organ involvement		
Lungs	44.4	20
Bones	24.4	11
Central nerve system	24.4	11
Nonregional lymph nodes	24.4	11
Soft tissue	20.0	9
Liver	13.3	6
Skin	13.3	6
Pleura	8.9	4
Others	2.2	1

At 24-months follow-up: 48.0% (n = 59) of patients were alive without active disease; 12.2% (n = 15) were alive with disease; 28.5% (n = 35) died as a consequence of the disease; 6.5% (n = 8) died due to other causes; and 4.9% (n = 6) of patients were lost to follow-up (failure to attend controls). 60.0% (n = 27) of patients with progression of the disease (local, regional, or systemic) died during the time of study.

Overall survival for the group of patients with early and locally advanced disease, contributed a total of 2,384 months of follow-up, time during which 29 deaths occurred. 

**Figure 1:**
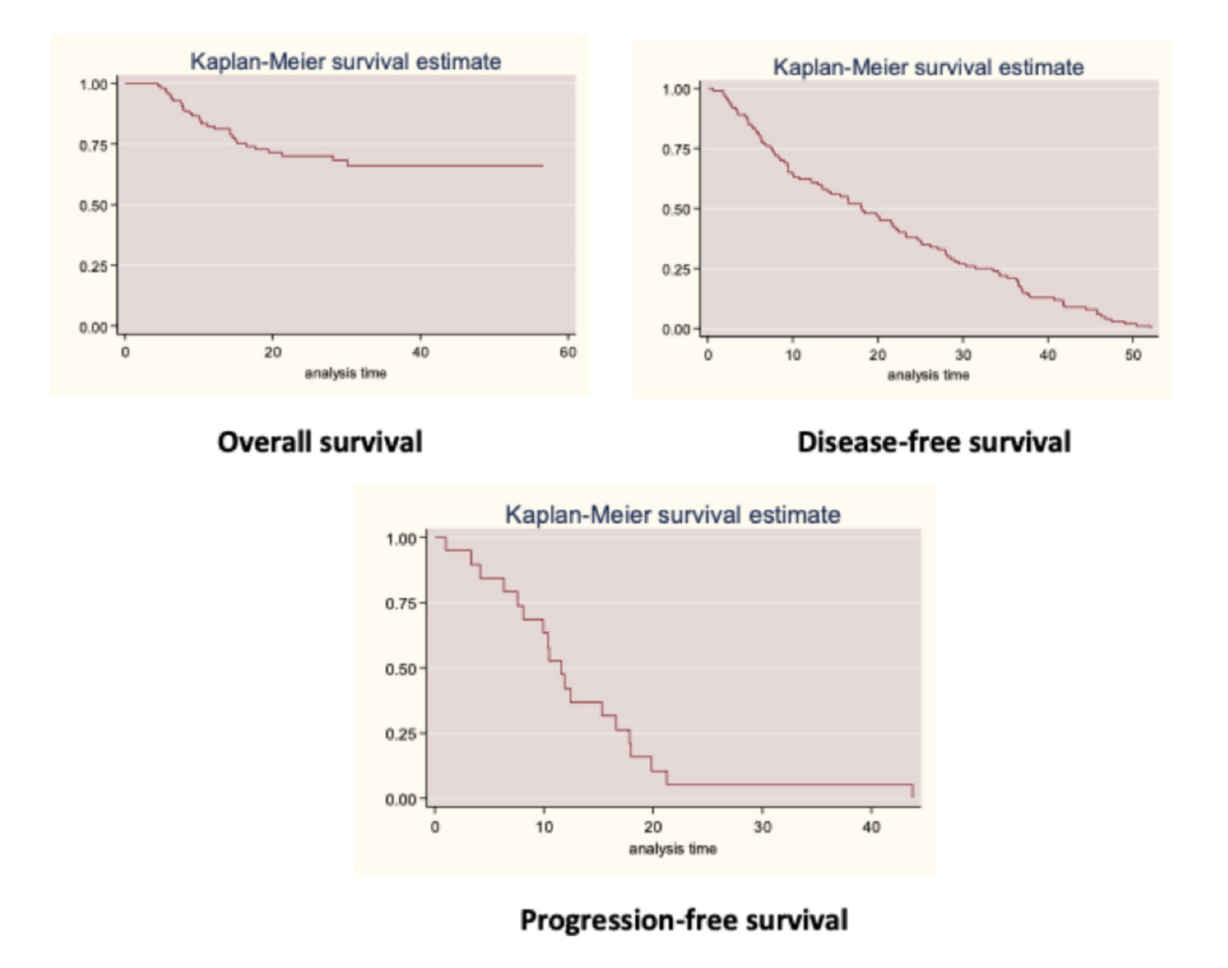
Overall survival, disease-free survival, and progression-free survival within the cohort of patients diagnosed with TNBC at the NCI-FBCU.

The time at risk had a median of 22.3 months (0.7-56.7 months). The incidence death rate was of 1.21 deaths per 100 patients / month (95% CI 0.84-1.8) (Figure 1). 

The analysis of EFS was performed in patients with early and locally advanced diseases, thus excluding from the analysis patients with metastatic disease; this group of patients contributed 2,051 months of follow-up, time during which 100 relapses occurred. The median EFS was 18.2 months. The time at risk had a median of 18.1 months (0.7-52 months). The incidence death rate was of 4.88 deaths per 100 patients / month (95% CI 4-5.9) (Figure 1).

The analysis of PFS in stage IV patients included 21 patients (two were excluded because stage IV was by skin and chemotherapy was ordered with a neoadjuvant intention). Patients contributed 253.2 months, time during which 19 progressions occurred. The median PFS was 11.6 months. The time at risk had a median of 10.5 months (0.4-43.8 months). Progression incidence rate was of 7.5 progressions per 100 patients / month (95% CI 4.8-11.8) (Figure 1).

**Figure 2:**
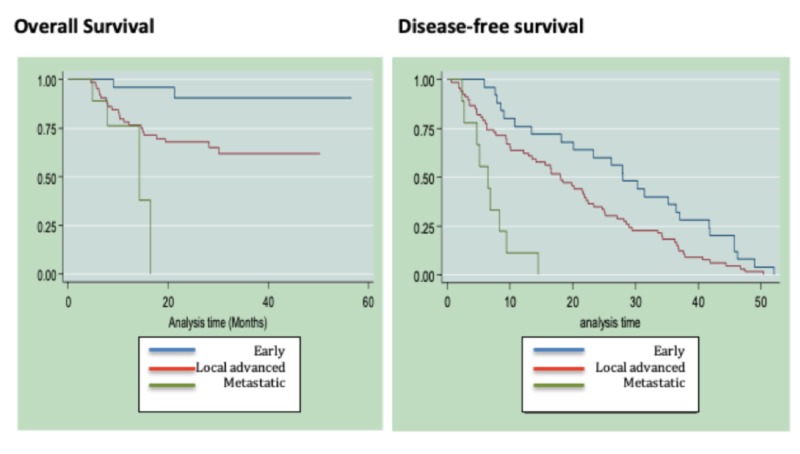
Overall survival and disease-free survival in relation to clinical stage within the cohort of patients diagnosed with TNBC at the NCI-FBCU.

There is no significant difference in OS functions and in EFS with respect to age over or under 50 years. A higher mortality rate was found as the clinical stage increased. The difference in survival functions is significant (Log-rank test: χ2 = 14.14 (2); p = 0.0009) (Figure 2). A higher recurrence rate was found as the clinical stage increased. The difference in OS functions is significant (Log-rank test: χ2 = 25.77 (2); p = 0.0009). As well, patients diagnosed with stage IV have a higher risk of death compared to patients with early and locally advanced tumors (HR 3.4, 95% CI 1.3-9-0; p = 0.013) (Figure 2).

## Discussion

Triple negative breast cancer is an entity that has become a therapeutic challenge for cancer centers and accounts for 15% of all breast tumors. It registers a more aggressive biological behavior, higher mortality rates, and risk of metastasis within the first three years after diagnosis [2].

The NCI is a reference cancer center at national level, and as Colombia is a developing country, most patients enter with locally advanced stages of the disease. For this cohort, specifically, 61.0% (n = 75) of cases were locally advanced tumors, and 18.7% (n = 23) were stage IV.

Unfortunately, the Institution's diagnostic protocol includes only four immunohistochemistry markers, and it is difficult from the administrative point of view that healthcare providers authorize additional markers, reason why only 7% of our patients have other IHC markers, and it is a limitation of our study to classify these tumors according to the classification described by Lehmann et al., as we do not have enough resources to do so [6].

Case series worldwide show that unlike luminal tumors where bone is the most common location of metastatic disease [10], in TNBC patients the most common sites for metastasis are lungs and brain [11-12]; which is very similar to that observed in this work, where lungs were the most common location of appearance of the disease at stage IV (47.8%) as well as for recurrence (44.4%).

Pathological complete response to neoadjuvant chemotherapy in this cohort is much lower (22.6%) than that registered in randomized clinical trials and meta-analyses ranging from 33% to 42% [13-16], which could be attributed to advanced stages of the disease, and in some cases due to problems with the authorization of medicines by the insurer. In this cohort, the pCR when using platinum regimens was of 31.5%, a very low value compared to results presented in the GeparSixto and CALGB 40606 [17-18].

In the German group’s study, GeparSixto administered neoadjuvant chemotherapy with paclitaxel, liposomal doxorubicin and bevacizumab, with or without weekly carboplatin, in 315 patients with TNBC, finding pCR in 53.2% of patients treated with carboplatin, compared to 36.9% of those who did not receive the medication (p = 0.005); reporting statistically significant differences in the two arms of the study, concluding that the addition of a carboplatin to neoadjuvant therapy with a taxane and/or anthracycline, significantly increases the rate of pCR in this group of patients [17].

Likewise, CALGB 40603 assessed weekly paclitaxel with or without carboplatin and/or bevacizumab followed by a dose of doxorubicin and cyclophosphamide, thus reporting an increase in pCR rate from 46% to 60% in favor of the study branch that received carboplatin (p = 0.018). In this study, pCR did not impact OS [18].

In the meta-analysis by Cortazar et al. [13], OS is reported in the subgroup of patients with TNBC with an HR: 0.16 (0.11-0.25) and EFS with an HR: 0.24 (0.18-0-33). In our study, OS was of 92% in patients who achieved pCR.

In this cohort, radical surgery was performed in a higher percentage, which is explained by the higher frequency of locally advanced tumors. In this same sense, the low percentage of immediate breast reconstruction draws attention, which is attributed mainly to administrative problems with health providers, availability of the plastic surgeon and little interest on the part of patients to carry out their immediate reconstructive procedure. Moreover, local recurrence was higher in patients taken to radical surgery, which is also explained by the advanced stage of their disease.

In our cohort, only two patients received adjuvant treatments with capecitabine, given that we still did not have results of the CREATE X study, which was published in June of 2017.

Masuda et al. reported in the subgroup of patients diagnosed with TNBC (n: 286) with residual disease, that the administration of six cycles of capecitabine as adjuvant treatment, positively impacted five-year event-free survival (69.8% vs. 56.1%) (HR 0.58, 95% CI 0.39-0.87), and 78.8% vs. 70.3% OS (HR 0.52, 95% CI 0.30-0.87) [19].

In our cohort, 28.5% of patients died due to the disease, and 12.2% are in active treatment for their metastatic disease. Lin et al. evaluated 12,902 women by comparing patients with positive-hormone-receptor tumors and patients with TNBC, reporting a worse OS in patients with TNBC, in a study published in 2012 (HR 2.72, 95% CI 2.39-3.10), and an increased risk of death in the first two years after diagnosis (HR 6.10, CI 95% 4.81-7.74) [20].

However, this risk decreases with time, with a 10-year relapse-free interval of 97% and a 15-year interval of 95%. Regarding relapse-free survival, it was of 91% and 83%, respectively [20-21].

Overall survival of TNBC is lower compared to other molecular subtypes. In luminal A tumors is of 92.5%, in luminal B HER2-positive is 90.3%, in pure HER2 (HER2-positive, negative-hormone-receptor) 82.7%, and in TNBC is of 77% [22]. In our cohort, disease progression appeared in 36.6% of cases, and mortality was higher in locally advanced and metastatic tumors. No connection was found with age, previous data that coincide with the work of Li et al. [23].

Breast cancer research is currently aimed at patients with TNBC, especially in the field of immunotherapy both in metastatic disease and in the neoadjuvant and adjuvant setting [24-26].

Among limitations of this work, we can find derivatives of the retrospective studies to establish causal relationships, and the difficulty of conducting follow-up on patients due to changes in contracting with other institutions. Therefore, the size of the sample would not allow subsequent statistical analyzes with multiple covariates and their association with the outcome.

## Conclusions

Triple negative breast cancer is a heterogeneous and aggressive disease with a worse OS rate and a higher risk of metastasis compared with other molecular subtypes, findings that coincide with the results of our cohort.
